# Adverse events, bone mineral density and discontinuation associated with generic alendronate among postmenopausal women previously tolerant of brand alendronate: a retrospective cohort study

**DOI:** 10.1186/1471-2474-11-68

**Published:** 2010-04-14

**Authors:** Daniel T Grima, Alexandra Papaioannou, Parisa Airia, George Ioannidis, Jonathan D Adachi

**Affiliations:** 1 Cornerstone Research Group Inc, 3228 South Service Road, Burlington, ON, L7N 3H8, Canada; 2 Center for Osteoporosis and Arthritis Research and Education (COARE), 1185 Rushbrooke Dr, Oakville, ON, L6 M 1H8, Canada; 3 Department of Medicine, McMaster University, 1200 Main St W, Hamilton ON, L8N 3Z5, Canada; 4 Division of Rheumatology and Department of Medicine, McMaster University, 1200 Main St W, Hamilton ON, L8N 3Z5, Canada

## Abstract

**Background:**

A rise in gastrointestinal (GI) adverse events (AEs) and a decline in bone mineral density (BMD) was observed in patients previously tolerant to brand alendronate shortly after generic versions were introduced in July 2005 to the Canadian market. The objective of our study was to quantify changes in AE rates and BMD scores, as well as associated alendronate discontinuation among patients before and after switch from brand to generic alendronate.

**Methods:**

A chart review of postmenopausal women 50 years of age and older between 2003 and 2007 was conducted in two specialized tertiary care referral centers. Patients on alendronate both before and after July 2005 were included. The change in the number of AEs, changes in BMD and associated alendronate discontinuation was compared before and after the switch from brand to generic alendronate.

**Results:**

301 women with an average age of 67.6 years (standard deviation (SD) = 9.5) had a total of 47 AEs between July 2003 and December 2007 that resulted in discontinuation of the medication. There was a significant increase in the rate of AEs per patient-months-at-risk from 0.0001 before to 0.0044 after October 2005 (p < 0.001). The most common AEs were GI in nature (stomach pain, GI upset, nausea, and reflux). In addition, 23 patients discontinued alendronate due to BMD reduction after January 2006. In these patients, BMD scores were significantly reduced from their prior BMD measures (change of -0.0534, p < 0.001 for spine BMD and change of -0.0338, p = 0.01 for femur BMD). Among patients who discontinued due to BMD reduction, BMD was stable in the period prior to January 2006 (change of -0.0066, p = 0.5 for spine BMD and change of 0.0011, p = 0.9 for femur BMD); however, testing for reduction after January 2006 in BMD measures (one-sided T-test) revealed there was a significant reduction in BMD scores for both anatomic sites (change of -0.0321, p = .005 for spine, change of -0.0205, p = 0.05 for femur).

**Conclusions:**

Patients who were previously stable on doses of brand alendronate experienced an increase in AEs causing discontinuation after introduction of automatic substitution to generic alendronate. In addition, reductions in BMD were observed in some patients who had stable BMDs before January 2006. Given the substantial increase in AEs, generic alendronate may not be as well tolerated as brand alendronate.

## Background

Osteoporosis is common in Canada affecting 16% of women and 6.6% of men over 50 years of age [1]. Despite the availability of a number of therapeutic options, many patients with fragility fracture do not undergo osteoporosis management and are at high risk for subsequent fractures [2-4].

Alendronate sodium has been extensively used for the treatment of osteoporotic patients in Canada. Generic alendronate versions were introduced in Canada in July 2005. As a result of automatic substitution implemented at the pharmacy level, over 80% of private and public plan patients were switched from brand to generic alendronate within two months. Typically, patients would not have been notified of the conversion. Shortly afterwards we noticed an increase in the frequencies of gastrointestinal (GI) adverse events (AEs) and bone mineral density (BMD) declines, in those who had previously been stable on brand alendronate.

The potential for an increased risk of GI AEs has been noted with brand versions of alendronate sodium, especially when taken incorrectly [5]. It is likely that similar risks are associated with generic versions, however clinical trials examining the GI tolerability of generic versions of alendronate compared to the original formulations are not available. The objective of this retrospective chart review was to quantify the number and type of AEs, and the proportion of AEs which led to discontinuation among patients before and after the switch from brand to generic alendronate.

## Methods

### Study design

Data were obtained from an analysis of patient charts from two specialized tertiary care referral centers in Hamilton, Ontario. Ethics approval for the study was not required as it was conducted as a self-audit of private practices. Patients were screened in alphabetical order from a list of all female clinic patients using the following inclusion criteria: age 50 years or older between 2003 and 2007, post-menopausal, confirmed osteoporosis and continuous treatment with alendronate sodium 10 mg daily or 70 mg once-weekly doses before and after July 2005. Data abstraction was conducted by one member of the clinical staff and was entered into a centrally maintained database using anonymous patient identifiers.

The following data were collected:

1. Visit dates

2. AEs noted within the patient chart as possibly related to the bisphosphonates under use. An initial list of AEs was developed from published clinical trials of original alendronate (alendronate sodium) and supplemented based on clinical experience with bisphosphonates therapy [6-9]. AEs captured included specific and general GI complaints (i.e., stomach pain/upset, GI upset, nausea, reflux, heartburn, bloating, constipation, diarrhea, rectal bleeding, bowel problems, perforated diverticulum, stomach ulcer), as well as unspecified trouble with apo-alendronate, chest pain, loss of appetite, general feeling of being unwell, anemia, rash, shortness of breath, bone pain, arthralgias, flank pain, and leg cramping.

3. BMD at femoral neck and vertebral sites. BMD testing was conducted annually in most patients. BMD measurements were made using a Hologic Delphi QDR-W machine.

4. Discontinuation and initiation of osteoporosis treatments.

5. Patient age

6. Concomitant use of calcium, vitamin D, estrogen, proton pump inhibitors, non-steroidal anti-inflammatory drugs (NSAIDs) and H2 receptor antagonists

### Analysis

The switch from brand to generic alendronate occurred at the pharmacy level in July 2005 but since prescriptions are usually prescribed for a three- month period, exposure to both brand and generic alendronate would be expected between July 1, 2005 and October 1, 2005. As some AEs in this period may have been related to brand alendronate, we treated AEs during this three-month period as applicable to brand alendronate, which could bias results against the hypothesis of greater AEs with generic alendronate.

To consider equal time for each exposure period, we selected two time periods: July 1, 2003 to September 30, 2005, and; October 1, 2005 to December 31, 2007. The number of AEs resulting in discontinuation of treatment was compared in the two study periods. To take into account the effect of duration of treatment on development of AEs, the rate of AEs was calculated per patient-months-at-risk. The rate of AEs per patient-months-at-risk was compared between the two study periods.

The total number of AEs and patient-time-at-risk were compared using chi-squared test. When evaluating BMD changes, January 1, 2006 was used as the threshold between periods as it may take a minimum of six months (from the time of switch) for changes in BMD to appear in bone scans. Therefore such changes in BMD observed between July 1, 2005 and January 1, 2006 would be most likely related to treatment with brand alendronate. Two analyses were conducted on BMD. First, for those visits at which discontinuation of alendronate was noted due to a decline in BMD, the BMD data were analyzed to confirm a decline in BMD, by comparing BMD at the alendronate discontinuation visit to the preceding BMD measurement. Since the differences in BMD measures between the two visits followed a normal distribution, a two-tailed paired t-test was employed for this comparison. Second, for those occurrences where two or more BMD measurements were available before January 2006, the stability of BMD was examined to test the possibility of the effect of aging on BMD differences. For this purpose, three consecutive BMD measures were selected: the two BMD measurements immediately preceding January 2006 and the first BMD measurement occurring after 2006. The change in the first two measures (both occurring before January 2006) and the change between the last BMD measure before January 2006 and the first BMD measure after January 2006 were calculated. To test stability of BMD scores in the two time periods, the average change in BMD scores was compared to 0 (no change). Again, since BMD differences between compared measurements followed a normal distribution and because significant reduction was only hypothesized, one-sided T-tests were employed.

The data was managed and the analyses were performed using SPSS version 16.0. Analyses were independent of the sponsor based on a data analysis plan established prior to study start.

## Results

**Figure 1:**
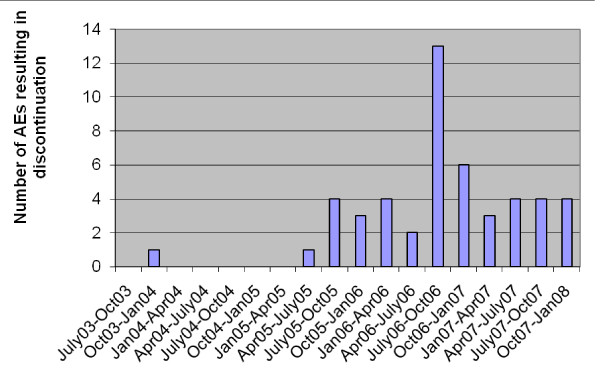
**Number of AEs resulting in discontinuation over the study periods (3 months interval)**. AE: adverse event.

A total of 301 patients were included in the study of AEs. Table 1 shows the baseline characteristics and the covariate distribution in this study population. Patients ranged in age from 50.62 to 89.23 years old, with a mean age of 67.6 (SD = 9.5) years at the time of the switch. Use of aspirin/NSAIDs, calcium, vitamin D, and estrogen was similar in the two time periods (Table 1). A summary of the type and frequency of observed AEs is shown in Table 2. The most frequent AEs were GI in nature (stomach pain, GI upset, nausea, and reflux). There were no deaths or laboratory AEs in the study. Figure 1 shows the number of AEs resulting in discontinuation over the study periods. The increase in the number of AEs after July 2005 is notable. The total number of patient-months-at-risk (total months patients were on treatment of interest) between July 1, 2003 and October 1, 2005 was greater than that between October 2005 and December 2007 (20,492 vs. 9,929) due to the greater number of discontinuations in the latter time period. The rate of AEs per patient was significantly higher in the second time period (0.0001 vs. 0.0044 per patient-months-at-risk). The rate was statistically significantly higher after October 1, 2005 (p < 0.001). Table 3 summarizes these results.

**Table 1:** Baseline characteristics of the study population

Characteristic	First Period (July 2003 to September 2005)	Second Period (October 2005 to December 2007)	Difference (p value of the test of difference)
Mean age (years)	65.6 (9.5)	67.6 (9.5)	2 (<0.0001)

NSAID use (%)	31%	25%	6% (0.08)

Vitamin D use (%)	81%	86%	5% (0.1)

Calcium use (%)	80%	75%	5% (0.17)

Estrogen use (%)	6%	9%	3% (0.16)

SD: standard deviation; NSAID: non-steroidal anti-inflammatory drug

**Table 2:** Frequency of different AEs in the study population

AEs	Frequency of occurrence	Percent of all AEs
Stomach Pain/Upset	16	22.86

GI Upset	11	15.71

Nausea	5	7.14

Reflux	5	7.14

Heartburn	4	5.71

Bloating	3	4.29

Constipation	3	4.29

Trouble with Apo-Alendronate	3	4.29

Chest Pain	2	2.86

Diarrhea	2	2.86

Loss of Appetite	2	2.86

Rectal Bleeding	2	2.86

Generally Unwell	2	2.86

Stomach Ulcer	1	1.43

Anemia	1	1.43

Rash	1	1.43

Perforated Diverticulum	1	1.43

Shortness of Breath	1	1.43

Bone Pain	1	1.43

Bowel Problems	1	1.43

Arthralgias	1	1.43

Flank Pain	1	1.43

Leg Cramping	1	1.43

Total	70	100.00

AE: adverse event; GI: gastrointestinal

**Table 3:** Total number of patients, years at risk and AEs leading to discontinuation

	July/03-Oct/05	Oct/05-Dec/07
Total number of patients	301	301

Number of AEs resulting in discontinuation	3	44

Number of patient months at risk	20492	9929

Rate of AEs per patient-months-at-risk	0.0001**	0.0044**

AE: adverse event; **Difference between two periods is significant at p < 0.001 level

**Figure 2:**
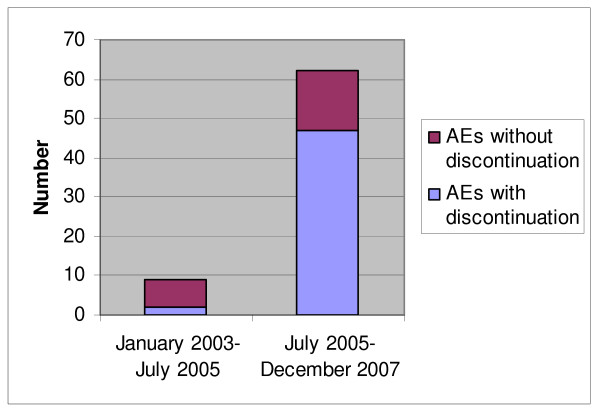
**Number of AEs with and without discontinuation in the two study periods**. AE: adverse event.

Figure 2 shows the proportion of AEs that resulted in discontinuation in the two study periods. Rate of AEs was greater in the second period when patients were on generic alendronate compared to in the first period when patients were on original alendronate. AEs were severe enough to warrant discontinuation of alendronate in over 21% of cases before October 1, 2005 and 79% of cases after October 1, 2005 (Figure 2).

Discontinuation of alendronate due to BMD reduction after January 2006 occurred in 23 patients. The average BMD score for those who discontinued alendronate after January 2006 was 0.756 g/cm^2 ^(SD 0.120) and 0.664 g/cm^2 ^(SD 0.130) for spine and femur respectively, while in their previous measurements these patients had average BMD scores of 0.813 g/cm^2 ^(SD 0.138) and 0.699 g/cm^2 ^(SD 0.129) for spine and femur respectively. This reduction in BMD scores was significant (p < 0.001 for change in spine BMD score and p = 0.01 for change in femur BMD score). Table 4 summarizes these results.

**Table 4:** Comparison of BMD measures (g/cm2) resulting in discontinuation to the previous measurement (n = 23 for spine and n = 19 for femur)

BMD Score	Measure after January 2006 resulting in discontinuation (g/cm^2^)	Prior measure (g/cm^2^)	Difference (p value of the test of difference)
Spine	0.756	0.813	-0.0534 (<0.001)

Femur	0.664	0.699	-0.0338 (0.01)

BMD: bone mineral density

**Figure 3:**
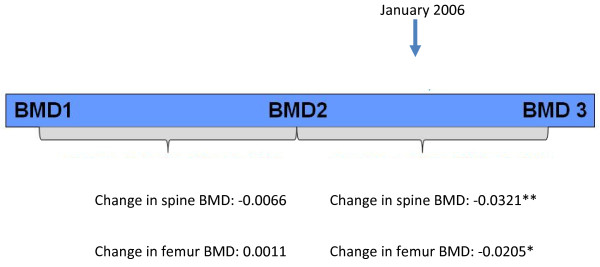
**Comparison of the change in BMD scores over time**. BMD: bone mineral density.

To test the possibility that the decline in BMD measurements in these patients were due to aging, we compared the stability of BMD before January 2006 and after January 2006. Among patients who discontinued due to BMD reduction and who had at least two other BMD measures before 2006 (n = 11 for spine and n = 9 for femur), we found that BMD was stable in the period prior to January 2006 (p = 0.5 for change of -0.0066 in spine BMD score and p = 0.9 for change of 0.0011 in femur BMD score) (see Figure 3). However, testing for reduction in BMD after January 2006 (one-sided T-test) revealed a significant reduction in BMD scores for both anatomic sites (p = .005 for change of -0.0321 in spine BMD, p = 0.05 for change of -0.0205 in femur BMD).

## Discussion

Many generic copies of alendronate sodium are available on the provincial formulary in Ontario, Canada, but not all of these formulations have been fully characterized in terms of therapeutic equivalence or safety/tolerability profile relative to original alendronate. Of particular relevance is the GI tolerability of the various formulations, as bisphosphonates in general have the potential to cause GI irritation and implications may exist for individualizing treatment. This study found that introduction of generic alendronate was associated with an increase in GI AEs leading to alendronate discontinuation and occurrences of discontinuation related to reduced BMD.

Oral bisphosphonates are poorly absorbed in the GI tract [10]. They have been associated with an increased risk of upper GI AEs, especially when taken incorrectly [5]. While the upper GI tolerability profile of the bisphosphonate, alendronate sodium (Fosamax) was similar to that of placebo in controlled clinical trials [6,11], generic formulations have not been studied as extensively and it is unclear whether they differ in GI tolerability from the original formulation [12,13]. Based on the belief that GI side effects, in particular esophageal irritation, result from events that occur prior to absorption of the drug into the GI tract, it has been postulated that the length of time required for a given formulation to disintegrate and dissolve may impact the tendency of the drug to cause irritation [12]. In vitro studies of various generic formulations of alendronate have demonstrated variations in disintegration and dissolution profiles as well as potential for esophageal bioadhesion, and have suggested that these differences may influence bioavailability, pharmacokinetics, efficacy, and tolerability [12,14-16].

In one study [12], several generic copies disintegrated two- to ten-fold faster than brand Fosamax. Other copies disintegrated at least five-fold slower than Fosamax. Yet another generic did not fall into either category but exhibited potentially large inter- and intra-lot variability. In another study [17], the mean disintegration times of the generic alendronate tablets ranged from 14 s to 342 s. The mean disintegration time of the Fosamax tablets ranged from 43 s to 78 s. Six of twenty-six generic tablets had very rapid disintegration times which were two to three times more rapid than Fosamax [17]. These results show that important differences may exist between Fosamax and its copies with regard to bioavailability, pharmacokinetics, and clinical efficacy and safety profiles. It is possible that rapid disintegration may increase esophageal drug exposure and possible toxicity leading to decreased upper GI tolerability. Slower disintegration may place the drug in contact with food or beverages severely reducing its bioavailability, reducing the efficacy as bisphosphonates [18]. Additional testing is warranted to evaluate the clinical safety and efficacy of these copies.

Our study also raises the possibility that disintegration profile led to an increase in AEs; the majority of which resulted in discontinuation. In addition, the BMD data indicate that differences in effectiveness between brand and generic alendronate may also exist, as 7% of patients (23 out of 301) discontinued generic alendronate due to significant reduction in their BMD scores, which had previously been stable. These results are supported by a recent analysis of Canadian administrative claims data, which found that the risk of discontinuation doubled in patients initiated with generic alendronate compared to patients started on branded alendronate [19]. We were unable to find published evidence that there is a common trend for discontinuation to be higher in patients using a generic version of a product; however the literature in this area is limited [20,21].

In addition, a recent chart review provides support to our findings. In a study by Ringe and Möller 2009, the generic alendronate was found to have lower persistence, greater GI AEs and lower increases in BMD compared to brand alendronate and risedronate. The chart review studied three contemporaneous cohorts of patients initiated on each of the three therapies [22].

Several limitations of the study are related to the retrospective and non-randomized design. To avoid preferentially selecting those patients who experienced AEs with generic alendronate, we screened all female patients in the clinic and selected all patients who were continuously treated with alendronate before and after July 2005. Socio-demographic differences may have been present, automatic substitution occurring more frequently in lower income patients with a greater number of co-morbidities. We were unable to test this hypothesis; however given the policy of automatic substitution for public plan patients, which include all patients over 65 years, this bias is unlikely.

With a retrospective chart review there exists the possibility that a systematic difference between the two periods could account for the changes in the outcome. To our knowledge, in the period of this study, no other changes that could have a negative effect on patients' BMD occurred. However, we are not able to completely rule out the possibility of period effect by factors not measured in this study. As with all measurements that occur over time, there is also the possibility of measurement error in BMD over time. However, it seems improbable that the error happened in a systematic manner that would bias against generic alendronate. Changes in BMD technology may have occurred at measurement sites during the study; however, most of the patients were studied using the same machine in most of their measurements. Such non-systematic measurement error likely introduced noise into the dataset which could have made detection of differences in BMD measures more difficult but it is unlikely to have biased the results.

In summary, among non-Fosamax formulations of alendronate, variation has been observed in terms of the rates at which they disintegrate and dissolve, and it has been suggested that this variation may correlate with variations in GI tolerability [12]. Several examples of mechanisms by which a causal relationship may exist have been reviewed [12,14,17], including the supposition that rapid disintegration could increase oral and esophageal mucosal exposure to active drug, thereby increasing the likelihood of irritation. Conversely, characteristics such as tablet shape and coating may delay esophageal transit and increase the risk of pill esophagitis, which may in turn be exacerbated by a formulation with a longer disintegration/dissolution time [12,14]. Currently, however, data directly evaluating the potential associations between variable disintegration profiles and degree of gastric irritation are scarce.

## Conclusions

Patients who were previously stable on doses of brand alendronate experienced an increase in AEs causing discontinuation after introduction of automatic substitution to generic alendronate. In addition, reductions in BMD were observed in some patients who had stable BMDs before January 2006. Our study suggests that there may be differences in both tolerability and effectiveness between brand and generic alendronate. Further study is warranted.

## Abbreviations

GI: gastrointestinal; AE: adverse event; BMD: bone mineral density; NSAID: non-steroidal anti-inflammatory; SD: standard deviation.

## Competing interests

The project was sponsored by research grants from the Alliance for Better Bone Health (Procter & Gamble Pharmaceuticals Canada Inc. and sanofi-aventis Pharma Inc.).

DG is a principle in and PA is an employee of Cornerstone Research Group, which received funding for this study from the Alliance for Better Bone Health, and also conducts analyses for other manufacturers of osteoporosis therapies.

AP - Research grant/Consultant/Speaker: Eli Lilly and Company, Merck Frosst, Amgen Inc, Procter & Gamble Pharmaceuticals, sanofi-aventis, and Novartis Pharmaceuticals Corporation. Clinical Trials: Eli Lilly, Merck, Novartis, Proctor & Gamble, sanofi-aventis.

GI did not have any competing interests.

JDA - Research grant/Consultant/Speaker: Amgen, Astra Zeneca, Eli Lilly, GlaxoSmithKline, Merck, Novartis, Nycomed, Pfizer, Procter & Gamble, Roche, sanofi-aventis, Servier, Wyeth and Bristol-Myers Squibb. Clinical trials for Amgen, Eli Lilly, GlaxoSmithKline, Merck, Novartis, Pfizer, Procter & Gamble, Roche, sanofi-aventis, Wyeth and Bristol-Myers Squibb. Stock: nothing to declare.

## Authors' contributions

All authors participated in the design of the study, as well as the analysis and interpretation of the findings. JDA secured the funding and was the primary investigator. AP was a co-investigator. DTG was project director. DTG, JDA and PA wrote the manuscript draft. All authors commented on and approved the final version.

## Pre-publication history

The pre-publication history for this paper can be accessed here:

http://www.biomedcentral.com/1471-2474/11/68/prepub
